# Quinine—Inaugural Thesis

**Published:** 1878-03

**Authors:** Thomas Northern


### QUININE.

INAUGURAL THESIS FOR THE DEGREE OF DOCTOR OF MEDICINE IN ATLANTA MEDICAL COLLEGE, MARCH 1, 1878.

BY THOMAS NORTHERN.

Quinine is a sulphate of quinia, one of the alkaloids of cinchona. It occurs in needles of a pearly and satiny appearance. It dissolves sparingly in water, but freely in alcohol and dilute sulphuric acid, and has an intensely bitter taste.

Its physiological action is exerted on the spinal cord, increasing its tone; on the stomach, increasing the secretion of gastric juice, but when long continued, it sets up gastric catarrh. In small doses, it increases the action of the heart and diminishes arterial tension. It arrests the amœliform movements of white corpuscles, aud prevents their migration. It diminishes the reflex function of the spinal cord, and is also regarded as an oxytocic. Perhaps most of these actions are the results of the influence exerted primarily upon the spinal cord.

Cinchonism is the term applied to the group of symptoms produced by the use of quinine, in which we have a sense of fullness in the head, a band-like feeling about the forehead, tinnitus aurium, giddiness and vertigo. Deafness occurs when large quantities are taken, and the hearing may be permanently impaired.

The maximum effect of quinine is obtained in about five hours, and elimination is effected, principally by the kidneys in about forty-eight hours.

The incompatibles of quinine are the alkalies and their carbonates, and the alkaline earths. It is also precipitated by the vegetable astringents, the tannic acid of which forms, with the quinia, a white, insoluble compound. Its synergists are the preparation of iron, arsenic and the mineral acids. It is antagonized therapeutically as an agent promoting constructive metamorphosis by mercury, the iodides and the salts of copper, zinc and lead.

Its bitter taste renders it very objectionable to patients, and on this account it is sometimes made into pills and coated with sugar; but when kept for a length of time, they become hard and insoluble, a condition very unfavorable for the administration of a medicine from which a prompt action is often so desirable. Liquorice is often used to hide the bitter taste, but *it* is objectionable to some patients. The most active form in which it can be administered is that of a solution in dilute sulphuric acid.

When, from irritability of the stomach, the quinina is not retained, we may administer it per anum. It is readily absorbed from the rectum, and it is thought by some to act more efficiently in this way; but most observers agree that a larger portion is required when introduced into the rectum. It sometimes happens that neither the stomach nor the rectum will retain the remedy; then, if the condition of the patient is such as to require the effect without delay, as when a paroxysm of pernicious intermittent is expected, we should resort to hypodermic injections. This last method has the advantage of acting more speedily than either of the others, and requiring a less quantity, but it is sometimes followed by abscesses, unless care is taken to have the remedy thoroughly dissolved, and the acids sufficiently diluted not to irritate.

In the use of quinia, we should not forget that some persons are very unpleasantly affected by quinine, even in small doses; therefore, the statement of patients in regard to this should not be disregarded. In such cases, small doses must be given, or some other remedy substituted. Fortunately, where we find this intolerance, we find that very small doses are curative.

The dose of quinine varies with the conditions that call for its use.

It is used in very many affections, but most effectually in malarial fever. The pathology of this disease is supposed to be seated in the spinal cord, and this calls for the tonic influence of quinine to restore tone to this depressed center, and the functions of organs dependent upon this nerve center for their nervous influence.

It was formerly the practice to prepare the system for this remedy by the administration of emetics, purgatives, etc.; but this preparatory course has been found not only unnecessary, but to some extent injurious, by setting up irritation of the stomach and intestines, which may be kept up by quinine, preventing its retention.

There are different opinions as to the time of administering the remedy in the different forms of intermittent. I believe it is considered best to begin in the sweating stage with doses of about five grains, and continuing until slight cinchonism is produced, and then continue such doses as may be necessary to keep up this effect till the time for the next paroxysm is past. If it were necessary to impress the system at once and with certainty, we might begin with doses of twenty-five, or even sixty grains. If we should fail to break up the paroxysm, we should begin in the same way to prevent the next succeeding paroxysm.

In remittent fever, we should select that period in which we have the most marked remission, and we will find that to occur between one and six o'clock in the morning. We should begin with a dose of ten to twenty grains, and continue with doses of from five to ten grains till cinchonism is produced slightly, and if you fail to secure this before the next paroxysm, the remedy may be continued through the exascerbation of fever. As in intermittent, we should lose no time in trying to bring about a remission with purgatives, emetics and diaphoretics, but should begin the remedy at once.

In the pernicious forms of intermittent and remittent fever, quinine is imperatively called for to prevent the recurrence of paroxysm, and it is in this that large doses are required and carried to the extent of quininism.

In typhoid fever, a disease depending upon a different poison and having its seat in a different part from that of malarial fever, quinine is never called for—in fact, its irritating property on the gastro-intestinal canal would contra-indicate its use; but in typho-malarial fever, it is called for in the early stage to subdue the malarial element, but should be suspended, if possible, before the glands of the intestine become involved.

In the anæmic condition following malarial fever, quinine, in combination with iron, is a valuable remedy. It is useful in all the diseases depending on this malarial poison, either primarily or secondarily. It is the best prophylactic of this poison, and after the intermittent form of fever has been subdued, quinine should be continued for three or four weeks to prevent its recurrence, to which there seems to be a special tendency on the seventh, fourteenth and twenty-first days, and on these days, especially, the system should be guarded.

When administered at the critical moment, it is said to arrest the inflammatory process. It is useful, also, in the eruptive fevers.

In all low states of the constitution, and in all diseases depending on a low constitutional condition, you will generally find quinine a valuable remedy, and when anæmia is present, it should always be combined with some preparation of iron.

